# Deep vein thrombosis causing abdominal wall varicosities

**DOI:** 10.1093/jscr/rjad004

**Published:** 2023-02-08

**Authors:** John H Norys, Kevin W Sexton, Hanna K Jensen, Matthew Deneke, Erin E Priddy

**Affiliations:** College of Medicine, University of Arkansas for Medical Sciences, Little Rock, AR, USA; Department of Surgery, Division of Trauma and Acute Care Surgery, University of Arkansas for Medical Sciences, Little Rock, AR, USA; Department of Surgery, Division of Trauma and Acute Care Surgery, University of Arkansas for Medical Sciences, Little Rock, AR, USA; Department of Internal Medicine, Division of Gastroenterology, University of Arkansas for Medical Sciences, Little Rock, AR, USA; Department of Radiology, Division of Vascular and Interventional Radiology, University of Arkansas for Medical Sciences, Little Rock, AR, USA

## Abstract

Deep venous thrombosis (DVT) is a common medical finding occurring in ~25% of hospitalized patients with roughly half of these patients experiencing post-thrombotic complications [Baldwin, Moore, Rudarakanchana, Gohel, Davies (Post-thrombotic syndrome: a clinical review. J Thromb Haemost 2013;11:795–805.)]. There are many associated complications of DVTs, including pulmonary embolism and lower extremity swelling; however, the occurrence of abdominal wall varicosities with DVT’s is rare [Baldwin, Moore, Rudarakanchana, Gohel, Davies (Post-thrombotic syndrome: a clinical review. J Thromb Haemost 2013;11:795–805.)]. The purpose of this case study is to rare presentation of abdominal vein varicosities as manifestation of DVT.

## INTRODUCTION

Deep venous thrombosis (DVT) occurs when blood clots form in the deep veins, namely the common and external iliac. Fractures of the lower limb are a common cause of DVT. The occurrence of DVT is relatively high, with ~0.2% of the general population and 25% of hospitalized patients experiencing complications annually [1]. Typically, thrombosis of a large vein in the abdomen, usually the inferior vena cava or portal vein, can lead to development of abdominal wall collaterals that contribute to abdominal varicosities visible on physical examination [2].

An important complication of DVT is post-thrombotic syndrome (PTS), which is a chronic consequence occurring in 50% of DVT patients [3]. This condition encompasses the manifestations of chronic vascular inadequacy that may result in development of lower extremity ulcers and collaterals in the abdominal wall leading to abdominal wall varicosities [3]. Importantly, the occurrence of abdominal wall varicosities is typically associated with PTS in cases of portal vein hypertension or inferior vena cava thrombosis [2]. This case study presents an example of PTS abdominal wall varicosities associated with lower extremity DVT of the left internal iliac vein.

## CASE REPORT

A 29-year-old male presented to the gastroenterology clinic with referral from the emergency department for evaluation of abdominal varicosities. The patient’s past medical history was significant for an emergency room (ER) visit 6 months prior in which he was evaluated for an abdominal mass thought to be an inguinal hernia upon initial physical examination. This was because of the presentation of a bulging lower abdominal mass in the pubic/inguinal area.

**Figure 1:**
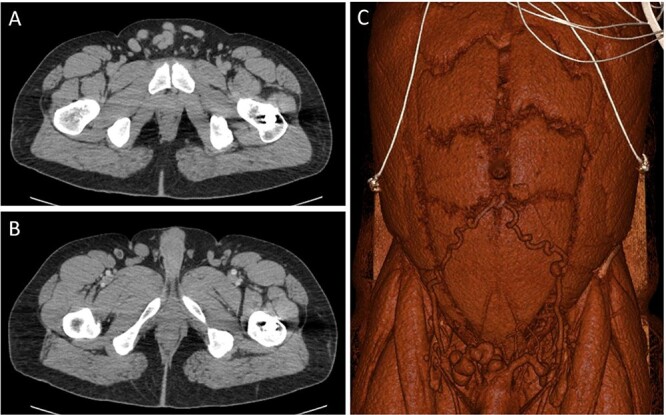
CT images through the pelvis show the dilated collateral veins (**A**) in the suprapubic region, initially thought to be a hernia. Solid white arrows (**B**) show the external pudendal vein confluences with the great saphenous veins. 3D reconstructed image (**C**) shows the dilated inferior epigastric and external pudendal veins, providing collateral flow around the stenosed left iliofemoral venous system.

An abdominal computed tomography (CT) in the ER indicated the mass was not a hernia but instead a cluster of abdominal varicosities (Fig. 1A and B), and the patient was referred to gastroenterology for evaluation. Other past medical history was significant for a motor vehicle accident (MVA) in 2019 in which the patient sustained left femoral and right tibia-fibula fractures requiring surgery. At that time the patient was not diagnosed with DVT; however, the patient reported significant edema in his left lower extremity since the MVA and swelling in his suprapubic region. Between the patient’s ER visit 6 months prior and his presentation on at the gastroenterology clinic, the patient consulted general surgery in which the physician expressed concern of the abdominal wall varicosities being a complication of his chronic DVT. In addition, the patient reported abdominal pain in his right upper quadrant (RUQ) consistent since his MVA in 2019; however, physicians believed this to be unrelated to his DVT.

The patient presented for imaging of his deep veins following his visit with GI for further evaluation of the abdominal varicosities. A lower extremity venous duplex examination was completed in which the patient was found to have chronic thrombosis of his left common femoral, femoral and popliteal veins. Importantly, no thrombosis was visualized in the patient’s inferior vena cava.

**Figure 2:**
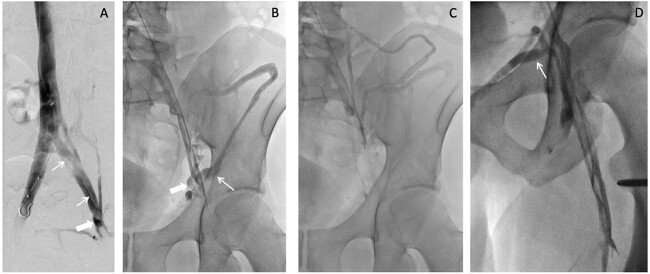
Iliocaval venogram **(A)** shows post-thrombotic changes including stenosis (thick white arrow) and synechiae (thin white arrows). Left external to common iliac venogram **(B)** shows collateral drainage pathway via the inferior epigastric vein (thick white arrow) to the contralateral external iliac vein and via the iliac circumflex (thin white arrow). Continued drainage **(C)** from the iliac circumflex into the iliolumbar and hemiazygos veins is shown. Saphenofemoral venogram **(D)** shows the sapheno-saphenous collateral drainage occurring via the external pudendal vein (thin white arrow).

A week after imaging, the patient presented to vascular and interventional radiology for further evaluation of the abdominal varicosities. Varicosities were found along the lower anterior abdominal wall due to formation of collaterals from the inferior epigastric vein. Iliocaval venogram indicated post-thrombotic changes including stenosis (thick white arrow) and synechiae (thin white arrows; Fig. 2). In addition, the patient presented with a small left internal iliac vein with stenosis distally viewed on ultrasound. The radiologist indicated that the left internal iliac appeared to ‘flatten’ under the right internal iliac indicating possible May–Thurner syndrome.

During the patient’s evaluation with GI, the patient did not present with issues related to portal hypertension such as splenomegaly, liver cirrhosis, or intraabdominal collaterals. Because of the lack of portal hypertension and IVC thrombosis, it is likely that the patient’s abdominal varicosities are due to chronic venous thrombosis of the lower extremities.

## DISCUSSION

An important aspect of this case is the presentation of abdominal wall varicosities initially suspected to be an inguinal hernia. Although these conditions can look similarly on physical examination, an important distinction is that inguinal hernias are often reducible [4]. In the instance when an abdominal mass is not reducible, imaging is required to differentiate the cause [4]. In this case, the patient presented with an abdominal mass in the pubic/inguinal area that was non-reducible during physical examination; imaging revealed the cause to be abdominal varicosities.

Although exceedingly rare, abdominal wall varicosities may be a complication of PTS in patients with chronic DVT as demonstrated by this case study. Becuase of the patient’s femoral fracture and subsequent edema following his MVA in 2019, it is likely the patient has been experiencing DVT and associated PTS since 2019. Importantly, there have been several case studies published indicating a relationship between DVT’s resulting in May–Thurner syndrome and abdominal varicosities and abdominal pain [5]. May–Thurner syndrome occurs when the right common iliac artery overrides and compresses the left common iliac vein [5]. Common manifestations of May–Thurner syndrome include lower abdominal pain, groin discomfort, itching, swelling and pelvic/abdominal varicosities [5]. Although still poorly understood, it is likely the varicosities develop in relation to May–Thurner syndrome due to deterioration of the venous walls and subsequent backflow of blood into abdominal veins, in this case the epigastric veins [5].

Treatment of abdominal varicosities in relationship to May–Thurner syndrome has also been documented. This involves ‘stab phlebectomy’ of the abdominal varicosities and sclerotherapy of the iliofemoral veins. Following this, a 4–6-week prescription of Aspirin and Clopidogrel must be followed for prevention of recurrent thrombosis with an antiplatelet or anticoagulant therapy being continued indeterminately [5].

Given the patients physical presentation, history of chronic DVT and radiology suggesting May–Thurner syndrome, the patient was treated for the abdominal varicosities following the regimen mentioned previously. In addition, there is significant reason to believe the patients RUQ pain is associated with his May–Thurner syndrome, and treatment will likely suppress the patient’s reported abdominal pain.

## AUTHORS' CONTRIBUTIONS

KWS conceived of the presented idea. JHN took the lead in drafting the case report. EEP took the lead on compiling relevant imaging. HKJ and MDD contributed to editing and drafting the manuscript. All authors discussed the case report and reviewed and edited the final manuscript.

## CONFLICT OF INTEREST STATEMENT

KWS has advisory positions and equity in Decisio Health, Inc.; Hoopcare, Inc.; hDrop Technologies, Inc. and an ownership of Datafy, LLC.

## FUNDING

None.
